# Case report of iatrogenic cerebral amyloid angiopathy after exposure to Lyodura: an Australian perspective

**DOI:** 10.3389/fnins.2023.1185267

**Published:** 2023-05-05

**Authors:** Claire Muller

**Affiliations:** ^1^Faculty of Medicine, University of Queensland, Brisbane, QLD, Australia; ^2^Department of Neurology, Royal Brisbane and Women's Hospital, Herston, QLD, Australia

**Keywords:** cerebral amyloid angiopathy, iatrogenic cerebral amyloid angiopathy, Lyodura, intracerebral hemorrhage, hemorrhagic stroke

## Abstract

**Background:**

Recently proposed diagnostic criteria for iatrogenic cerebral amyloid angiopathy (iCAA) have sparked increased recognition of cases across the globe. Whilst these patients tend to have a tumultuous course, much like sporadic CAA, there is a high degree of variability. What is unique in this case is the breadth of clinicoradiological data available, including handwritten surgical notes from 1985. In retrospect, early imaging changes of what would ultimately lead to profound morbidity, were apparent 30 years after inoculation with cadaveric dural tissue.

**Aim:**

In this case study we examine the clinicoradiological features of a case of probable iCAA and draw awareness to the presence of this disease in Australia.

**Methods:**

This case was admitted under the care of the author at the Royal Brisbane and Women's Hospital (RBWH). Clinical details and data were gathered during the patient's care and consent for publication provided by the enduring power of attorney.

**Results:**

This 56-year-old female presented in 2018 with left hemiparesis, neglect, and dysarthria secondary to a large right frontal lobe intracerebral hemorrhage (ICH) without an underlying macrovascular cause. MRI brain demonstrated diffuse superficial siderosis assumed related to previous surgical interventions during the mid-1980s for a Chiari malformation and cervical syrinx. There was evidence of extensive white matter disease, discordant with her lack of cerebrovascular disease risk factors. Brain biopsy confirmed CAA. Archived surgical notes confirmed exposure to Lyodura in 1985 and 1986. Two decades of MRI data were available for review and illustrate the evolution of CAA, from normal post-operative findings to marked and unrecognized abnormalities 4 years prior to her first ICH.

**Discussion:**

This is the first Australian case of probable iatrogenic CAA (iCAA) to have such extensive documentation of clinicoradiological evolution. It demonstrates the aggressive course iCAA can take and provides insights into early disease manifestations, relevant to the more common sporadic cases. A brief review of the history of commercial cadaveric tissue use in Australia highlights enormous changes in medical practice over the last 50 years. Awareness within Australia should be raised for this clinical phenomenon, and cases collated to contribute to the growing international pool of evidence.

## 1. Introduction

In the last 5 years there has been a surge in the number of cases of presumed iatrogenic cerebral amyloid angiopathy (iCAA) reported. Following pivotal presentations at the 2020 International Cerebral Amyloid Angiopathy Association (ICAA) Conference, case finding increased exponentially with more than 50 cases worldwide now published (Kovacs et al., 2016; Nakayama et al., 2017; Hervé et al., 2018; Jaunmuktane et al., 2018; Banerjee et al., 2019, 2022; Giaccone et al., 2019; Hamaguchi et al., 2019; Tachiyama et al., 2019; Raposo et al., 2020; Caroppo et al., 2021; Yoshiki et al., 2021; Kellie et al., 2022; Milani et al., 2023). Diagnostic criteria for iCAA have recently been proposed, noting the transmission potential of cadaveric CNS material (Banerjee et al., 2022). Lyodura was a commercially available lyophilized, irradiated human dura mater sourced postmortem. It was approved for use in Australia by the Therapeutic Goods Administration (TGA) in 1972 and withdrawn in 1987, after the recognition of the first case of Creutzfeldt-Jakob disease (CJD) linked to its use (Centers for Disease Control (CDC), 1987). Brooke et al. attempted to estimate the prevalence of Lyodura use across Australia based on surveys of the Neurosurgical Society of Australasia and the Royal Australasian College of Surgeons in 1995 and 2001 respectively (Brooke et al., 2004). Additional information was gathered from the manufacturer, B Braun Melsungen, leading to an estimate of 2,208–2,478 grafts being used in Australia from 1972, but predominantly between 1982 and 1986 (Brooke et al., 2004). It was and remains an insurmountable feat to trace batches of Lyodura to the subsequent CJD cases and the amount, size, and contents of boxes of graft sheets varied. They were ideal for surgery owing to their long shelf life and ease of use—a simple defrosting process in warm water in the operating theater. Based on acceptable surgical practice at the time, it is also feasible that multiple patients would receive grafts from the same box and batch. Similarities can be drawn with the use of cadaveric human growth hormone (c-hGH) used in the Australian Human Pituitary Hormone Program (AHPHP) active within the same period but suspended and investigated in 1985 for suspected transmission of CJD (Koch et al., 1985). The subsequent 1994 Federal Government Inquiry, led by Professor Margaret Allars, (Australia. Inquiry into the Use of Pituitary Derived Hormones in A, 1994) revealed many mistakes across the program, not least of which was the absence of informed consent being gained from donors and recipients of cadaveric material. Similarly, it does not seem to have been standard practice between 1972 and 1987 to inform patients of the use of Lyodura. Legislation of informed consent practices was still relatively novel in developed countries, including Australia, the US, and the UK (Skene and Smallwood, 2002; Bazzano et al., 2021).

Governance and regulation of the removal of cadaveric pituitary glands in the AHPHP was problematic and mortuary attendants would earn a financial incentive of 2 Shillings per pituitary gland removed (Australia. Inquiry into the Use of Pituitary Derived Hormones in A, 1994), the equivalent to about $50 AUD in 2023. Furthermore, dural tissue sources could not be easily traced to allow source control as Lyodura batches would include tissue from a mixture of cadavers (Centers for Disease Control (CDC), 1987). As to the dural tissue treatment, it was thought that the lyophilization and gamma irradiation process would be sufficient to remove any pathogenic agents, though this was ultimately insufficient to stop prion disease and it would be decades before the transmission of Amyloid-β (Aβ) would be hypothesized.

The plausibility of human transmission of Aβ has evolved from several key steps of logic, starting with the discovery of histopathological evidence of both Alzheimer's disease (AD) and CAA on autopsy of 8 individuals who died from iatrogenic CJD (iCJD) (Jaunmuktane et al., 2015). All cases had received c-hGH, and none had a genetic predisposition to AD or CAA based on Apolipoprotein E (ApoE) genotyping. By 2017, various neuropathology case series had demonstrated the presence of Aβ postmortem in those that had developed iCJD secondary to either cadaveric human growth hormone (c-hGH) or cadaveric dural transplant (Jaunmuktane et al., 2015; Frontzek et al., 2016; Hamaguchi et al., 2016; Kovacs et al., 2016). A further autopsy study of 5 individuals exposed to c-hGH who did not develop iCJD, demonstrated seeding of Aβ and pathogenesis of CAA could occur in the absence of CJD (Ritchie et al., 2017);

In 2018, Purro et al. were able to access archived cadaveric pituitary glands and demonstrate not only the presence of substantial amounts of Aβ but also to seed the formation of CAA in intracerebrally inoculated mice, with a latent pathogenesis that did not occur in controls and was not secondary to the procedure alone (Purro et al., 2018). Aβ could be independently transferred and pathogenic.

Moving from the laboratory bench to the bedside of an affected patient, this case report provides an opportunity to examine the gradual and initially subtle evolution of iCAA over 40 years. It illustrates the insidious development of the disease and the subsequent significant morbidity.

## 2. Case description

This 56-year-old woman was admitted under the care of the author at a quaternary metropolitan hospital (RBWH) in 2018 after presenting with acute dense left hemiparesis, dysarthria, and neglect. Her blood pressure on presentation was 119/76. Initial CT imaging, including angiography revealed a large right frontal lobe intracerebral hemorrhage (ICH) and no underlying macrovascular lesion.

Her clinical history was gathered via her next of kin, medical records from the Queensland public health system and her Primary Care Physician. She was a Sicilian Australian non-smoker, non-diabetic, of healthy weight, with no hypertension, a healthy cholesterol profile (without treatment) and no family history of dementia, stroke, or ischaemic heart disease. She had no past medical history of any previous traumatic brain injury, stroke, transient focal neurological event (TFNE) or cognitive impairment. There was an ill-defined period of right upper limb paraesthesia in 2014 which was attributed to cervical radiculopathy considering concomitant pain.

Between 2016 and 2018 she was commenced on Aspirin, Enalapril and Rosuvastatin by a Primary Care Physician in response to incidental MRI findings of chronic small vessel ischaemia but she had not met criteria for primary or secondary stroke prevention at that time and those medications were ceased following this first admission with ICH in 2018, during which her average systolic blood pressure was 100 mmHg.

Formal digital subtraction cerebral angiography on Day 2 of her first admission was unremarkable.

**Figure 1:**
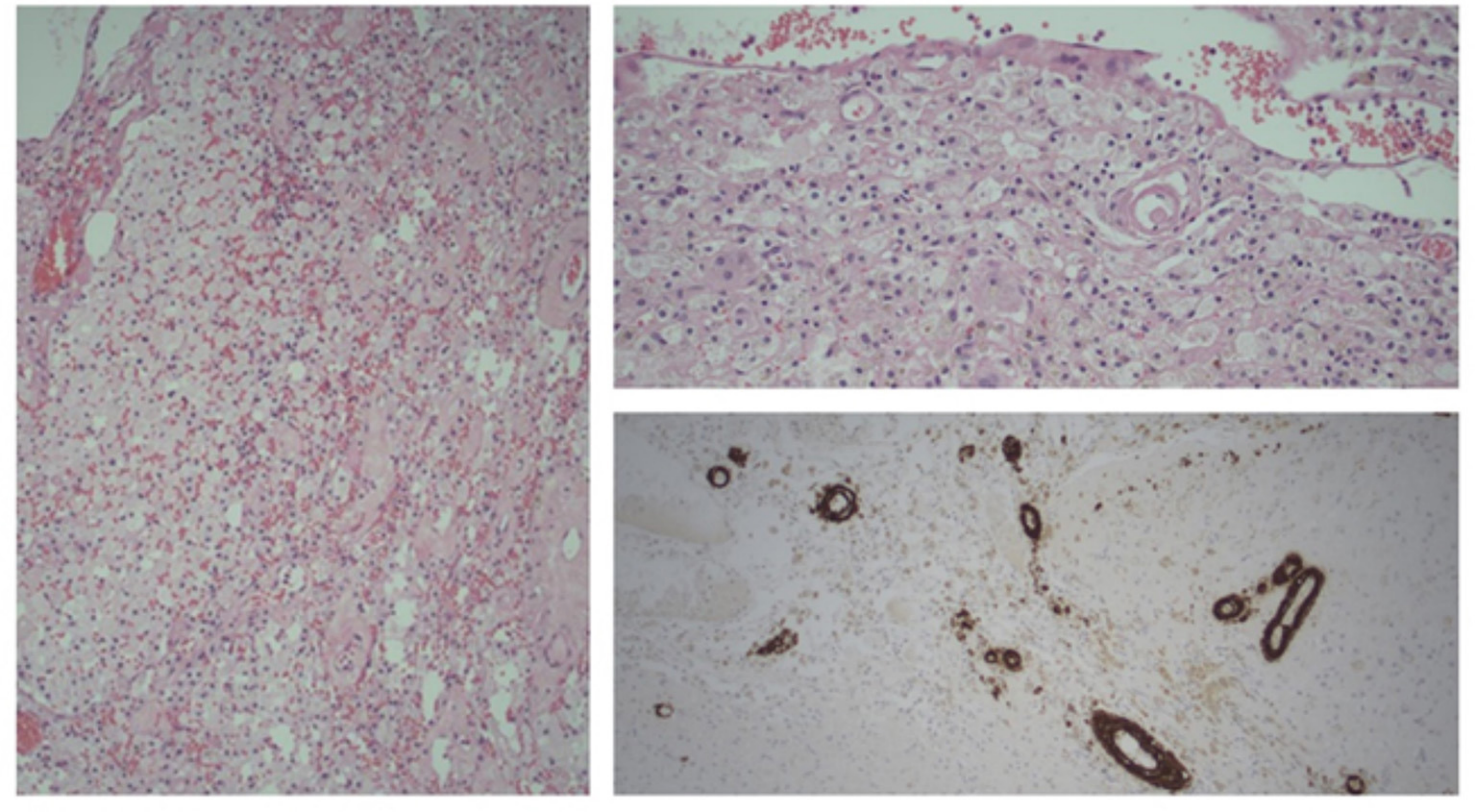
Brain biopsy. Old infarct with foamy macrophages and giant cells; blood vessels with hyaline thickening (H&E, Left 100x, top right 200x magnification). Bottom right, Beta amyloid positivity in vessel walls (IHC, Beta amyloid; 100x magnification).

MRI brain demonstrated multifocal and bilateral cortical superficial siderosis (cSS) and periventricular white matter hyperintensities (WMH) with some confluence but sparing of the basal ganglia and brainstem. Marked cerebellar siderosis was noted and initially thought related to a previous posterior fossa decompression for a Chiari malformation and cervical syrinx, a procedure she had undergone in 1985, and revised in 1986 at the RBWH. Extensive investigation for cerebral vasculitis was negative. She was managed in the acute stroke unit and stabilized for inpatient rehabilitation. Her initial improvement over several weeks was interrupted by a recurrent ICH. Over the subsequent 3 months she suffered 3 further ICHs in new locations, at near monthly intervals. Subsequent to the second ICH she underwent a brain biopsy which revealed CAA (Figure 1).

A genetic panel for CAA variants (APP, CST3, ITM2B, PRNP, TTR) was negative. Her ApoE genotype was ε3/ε3, conveying no predisposition to CAA or AD.

Between 2018 and 2023, the patient received 24 h supported care at her own residence after months of inpatient and outpatient formal rehabilitation. Her short-term memory was severely impaired, but some quality of life was garnered with her speech being largely intact, the capacity to stand transfer with assistance, and using an electric wheelchair outside the home. She was briefly re-admitted in 2019 for focal seizures with secondary generalization which ceased with regular Levetiracetam. Her course was further complicated by osteoporosis from prolonged immobility, currently managed with Bisphosphonates, and chronic pain owing to spasticity and radiculopathy.

Whilst there had been little improvement since 2018, she had not suffered a further ICH until February 2023, at which point she developed a new right sided hemiparesis and abulia. Repeat CT imaging demonstrated a new and large volume ICH in the left medial frontal and parietal lobe. She remains an inpatient and has made minimal improvements, with rare single word outputs, quadriparesis but intact alertness and swallow maintaining an unmodified diet with full assistance.

In 2021, the patient's archived medical records had been retrieved and documentation of the use of Lyodura was confirmed. Lyodural grafts were applied to the cranio-cervical junction in two separate neurosurgical procedures in 1985 and 1986.

**Figure 2:**
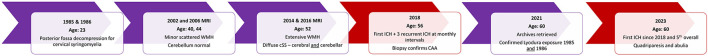
Clinical and radiological timeline overview.

**Figure 3:**
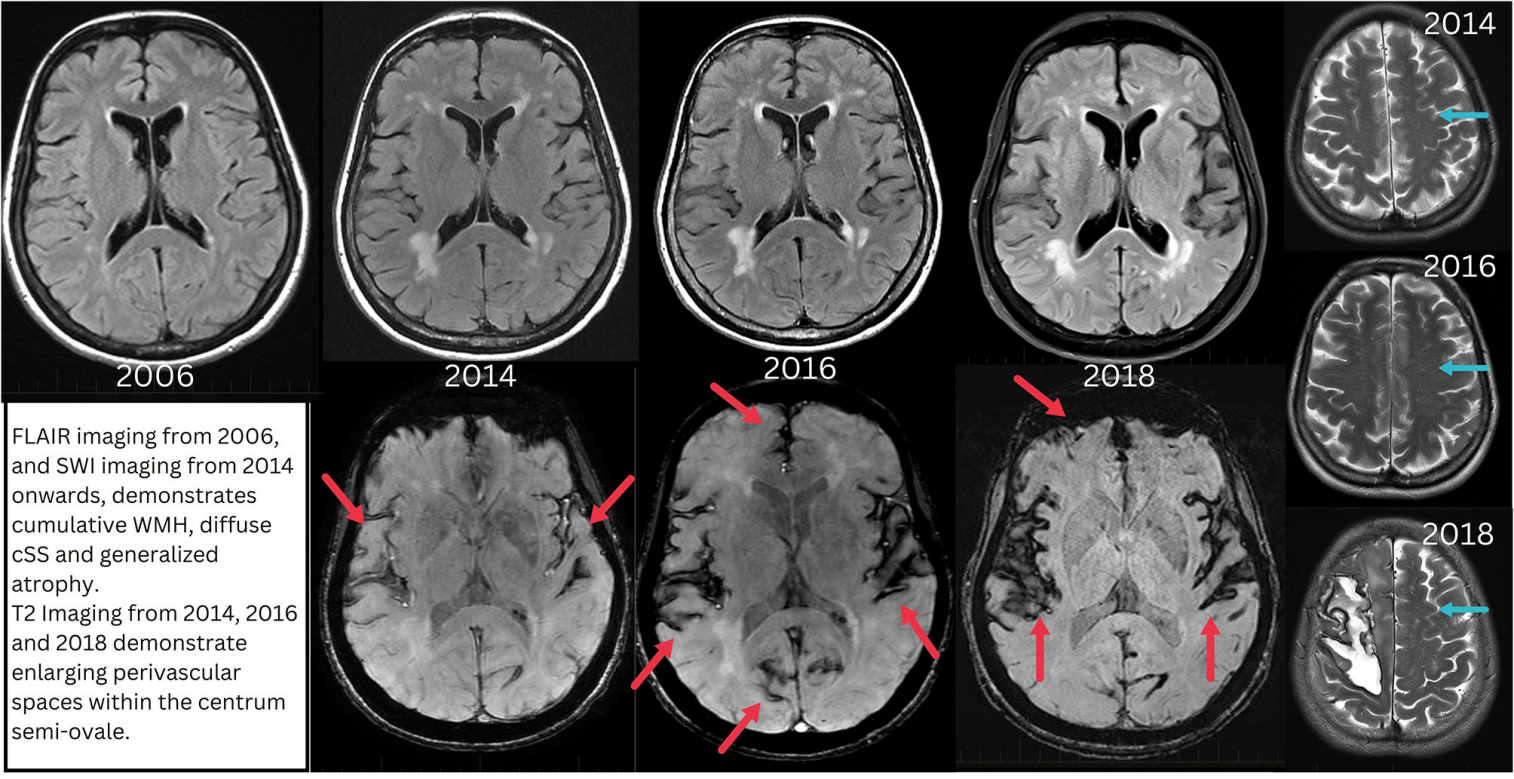
Progressive evolution of the imaging features of CAA between 2006 (20 years post-exposure) and 2018 (33 years post-exposure) when the first ICH occurs. Top row demonstrates progressive periventricular white matter hyperintensity development. Red arrows indicate areas of cortical superficial siderosis. Blue arrows indicate enlarged perivascular spaces within centrum semi-ovale.

**Figure 4:**
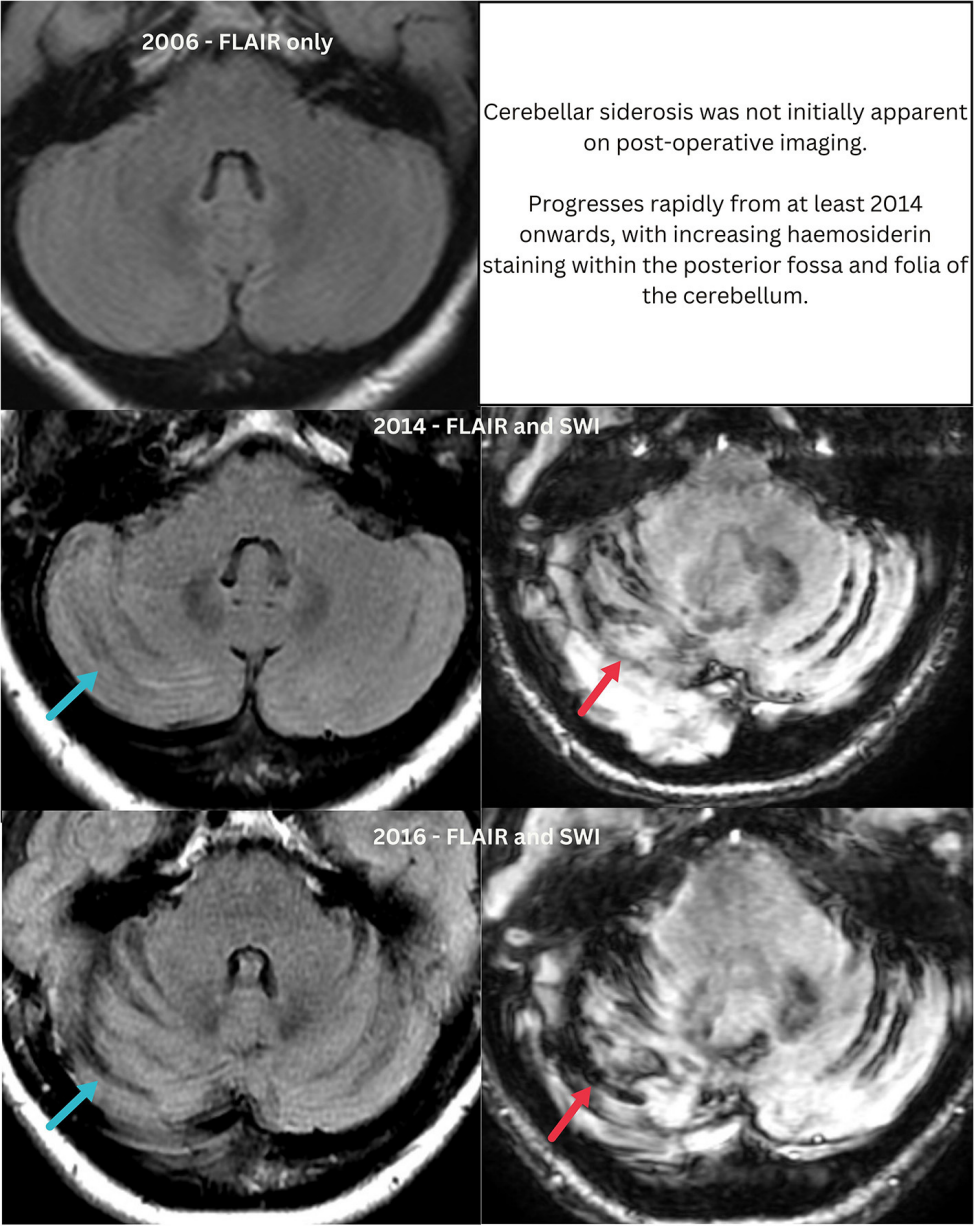
Progressive changes within the posterior fossa, mistaken for post-operative change as scans from 2014 were not correlated with prior imaging. Blue arrows indicate enlarged cerebellar folia, apparent from 2014 onwards. Red arrows indicate superficial siderosis which progresses from 2014.

Intensive investigation allowed collation of nearly two decades of MRI data for review (Figure 2). These scans demonstrated a marked progressive increase in periventricular WMH and multifocal cSS remote from the surgical site from 2014 onwards, disproportionate with age, vascular risk factors and past neurosurgery (Figures 3, 4). Notably, enlarged cerebellar folia and siderosis was not apparent until 2014, i.e., it was not post-operative as previously assumed in radiology reports from 2014 onwards.

In further detail, MRI results were available from 2002 to 2018 and CT imaging from 2012 to 2023. All MRIs were performed on a 1.5 Tesla machine up until 2018, at which point images were acquired with 3 Tesla field strength. MRI from 2002 and 2006 shows a normal cerebellum on T1 and T2 sequences without the enlarged cerebellar folia present from 2014 onwards. In 2014 and 2016, MR sequences included susceptibility weighted imaging (SWI) in addition to T1, T2, FLAIR, DWI, ADC map, 3D T1 FIESTA, and T2 fat saturation. In accordance with the STRIVE criteria (Wardlaw et al., 2013) the WMH is periventricular with no involvement of the basal ganglia or brainstem and consistent with a Fazekas grade 2. There are <10 enlarged perivascular spaces (EPVS) within the basal ganglia and 20 within centrum semi-ovale (CSO) in 2014 and 2016. There are no lacunes, cerebral microbleeds (CMB) or DWI lesions. With regards to cSS, this is notable in 2014 throughout the cerebellar folia, along bilateral sylvian fissures and the left cerebral convexity. By 2016 this has increased to involve further temporal cortex and the medial frontal lobe on the right and thus a high multifocality score of 4 based on the grading scale proposed by Charidimou et al. (2017).

By 2018, the 3T MRI (T1, T2, FLAIR, DWI, ADC, SWI) demonstrates increased confluence of WMH (Fazekas grade 2), with ongoing sparing of the basal ganglia and brain stem. EPVS have increased to 20 within the basal ganglia, but to more than 40 within the left CSO, the right being obscured by the acute ICH. The cSS remains similar to 2016 and there are still no CMB.

## 3. Discussion

This case of iCAA meets the proposed diagnostic criteria (Banerjee et al., 2022) and demonstrates the fulminant course the disease can take. Though the age at presentation is older than many published cases, the latency from exposure to symptoms is much the same at 33 years. The presentation with ICH, subsequent recurrences and homozygosity for the ε3 ApoE allele is also consistent with iCAA literature to date (Banerjee et al., 2022).

At the point of presentation with ICH, the patient would have met the Boston 2.0 criteria for sporadic CAA. However, florid imaging abnormalities were apparent at the age of 52 and likely to have evolved over greater than 2 years, making it highly likely the disease was present prior to the age of 50, the current cut off for the Boston criteria (Charidimou et al., 2022).

In this case, it is clear in retrospect that cerebral pathology separate to post-operative changes was developing over the years but did not prompt further investigation beyond considering the patient's risk factors for chronic small vessel ischaemia.

The increasing haemosiderin staining within the posterior fossa and the supratentorial subarachnoid spaces at the sylvian fissures was noted in MRI reports between 2014 and 2016 but assumed to be post-operative as no comparison had been made to prior images. In Australia there are multiple commercial and public health providers of MRI and the patient had imaging across at least 4 of these. There is no continuity between these radiology providers unless specifically arranged and therefore the emergence of new changes was not detected. The posterior fossa changes are notable as cerebellar siderosis is an emerging novel marker of CAA in both hereditary and sporadic forms (Koemans et al., 2022) and not specific to iCAA.

It remains to be seen whether iCAA cases follow an evolution in keeping with sporadic counterparts or is there something unique about the pathophysiology of CAA in the context of the introduction of exogenous Aβ to a milieu not primed to receive exogenous Aβ subtypes. Supporting the plausibility of this hypothesis is that most iCAA cases, present included, do not possess the ApoE alleles that would put them at risk of developing CAA. In a review of 23 cases of iCAA, ApoE genotyping showed all had at least one ε3 allele and two thirds were homozygous for ε3 (Banerjee et al., 2022). This is in contrast the higher rates of ε2 and ε4 alleles seen in sporadic CAA (Biffi et al., 2010). Alternatively, given the emerging evidence of sporadic CAA resulting from impaired or abnormal perivascular clearance systems in the CNS (Greenberg et al., 2020), it is possible that surgical procedures correlated with iCAA, disrupted these systems. Several case studies have linked early-onset CAA with a history of significant head trauma in preceding decades, but these reports are confounded by poor access to archived surgical notes to determine if cadaveric dural patches were used, as was common practice for penetrating head injuries (Oblak et al., 2022).

Given the prolonged latency from Lyodura exposure to development of CAA, it is likely more cases will be hidden across Australia. In this case it was 33 years from exposure to clinical recognition, in keeping with the latency range of 25–46 years previously reported (Banerjee et al., 2022). Given the period in which Lyodura was available (1972–1987) it could be anticipated that cases will continue to arise with acute clinical presentations over the next 10 years, and subsequently diminish if cadaveric tissue was causative.

In 1996, B Braun Melsungen ceased the production and sale of all Lyodura implants and there are currently only synthetic and bovine dural graft products approved for use in Australia by the TGA.

Overall, despite the spectrum of disease, a distinct phenotype of iCAA seems to be emerging in the literature. Thus far, cases appear to follow a tumultuous course with higher rates of ICH recurrence, seizures, and rapid cognitive decline, when compared to sporadic CAA (Banerjee et al., 2022). However, the phenomenon of iCAA remains relatively unknown to the broader medical community in Australia and highlighting these cases has bearing on whether or not patients will be referred on to a sub-specialist to manage their condition, even if education and explanation is the only treatment available. A larger cohort study is needed to examine this disease further and understand the natural history.

## Data availability statement

The original contributions presented in the study are included in the article/supplementary material, further inquiries can be directed to the corresponding author.

## Ethics statement

Ethical review and approval was not required for the study on human participants in accordance with the local legislation and institutional requirements. Written informed consent was obtained from the participant/patient(s) for the publication of this case report.

## Author contributions

CM is the sole author of this case report and completed all of the work necessary to compile the information required after acquiring written informed consent to publish and present de-identified material relating to the patient.
